# Spontaneous renal rupture caused by factor VII deficiency: A case report

**DOI:** 10.1097/MD.0000000000036130

**Published:** 2024-05-03

**Authors:** Chao Yang, Mingjun Luo, Langlang Li, Qizhi Yang

**Affiliations:** a Department of Urology, 302 Hospital of China Guizhou Aviation Industry Group, Anshun, Guizhou, China.

**Keywords:** case report, hemophilia, perirenal hematoma, spontaneous renal rupture, urinary extravasation

## Abstract

**Rationale:**

Spontaneous renal rupture is an uncommon disease, it usually occurs after upper urinary calculi-related operation treatment or renal tumor. This disease caused by factor VII deficiency has rarely reported.

**Patient concerns:**

A 49-year-old woman came to our hospital with on the left flank pain and gross hematuria that had persisted for 10 days. The patient had no recent history of waist and abdominal trauma or surgical history recently.

**Diagnoses:**

An outside computed tomography (CT) examination revealed left renal rupture before arriving at our hospital, but she was not treated. Further laboratory examination revealed that the patient condition was turned out to be hemophilia caused by factor VII deficiency.

**Intervention:**

We have used both internal and external drainage methods, and supplemented with coagulation factor.

**Outcome:**

After 9 months of follow-up, it was observed that the left renal hematoma and urinary extravasation was completely absorbed.

**Lessons:**

Spontaneous renal rupture for hemophilia is a clinical emergency. When spontaneous renal rupture is associated with abnormal coagulation function, and the coagulation function cannot be corrected by conventional treatment, the possibility of hemophilia needs to be considered, and the type of hemophilia needs to be further defined. This case indicates a successful resolution of spontaneous renal rupture, it can provide guiding value for our clinical practice.

## 1. Introduction

Spontaneous renal rupture is an uncommon clinical disease, according to the location of the rupture, it often leads to perirenal hematoma or urinary extravasation.^[1]^ These patients show fierce pain or gross hematuria, because of potential complications, there the need for urgent intervention is required. In the case of contralateral kidney lesions, there is a risk of acute renal failure occurring. The treatment methods for renal rupture are varied.^[1–3]^ The spontaneous renal rupture has been reported in many literature before, but it caused by hemophilia has hardly been reported. The report describes a rare case of left renal rupture caused by hemophilia, furthermore, the patient also had some atrophy of the right kidney. We reported the treatment process of this patient, with the aim of providing a reference opinion for the treatment of similar clinical cases.

## 2. Case presentation

A 49-year-old female patient presented to our hospital with left flank pain and gross hematuria persisting for 10 days, she did not any fever or chills. Before admission, the patient underwent abdominal computed tomography (CT) scan at an outside hospital, which revealed a left renal rupture, there were perirenal hematoma and urinary extravasation, there were also right renal atrophy and double kidney calculi, but she did not receive any treatment. The patient had no history of waist and abdominal trauma or surgical history recently, the physical examination showed obvious tenderness in the left renal region and vital sign monitoring was stable. Based on the laboratory tests that were completed, the results are as follows: blood routine; white blood cell counts is 7.51 × 10^9^/L (normal range, 3.5–9.5 × 10^9^/L), hemoglobin level is 51g/L (normal range, 130–160 g/L), renal function; urea is 18.9 mmol/L (normal range, 3.1–8 mmol/L), creatinine is 515 µmol/L (normal range, 57–97 mmol/L), coagulation function; prothrombin time is 42.20S (normal range, 10–14S), international standardized ratio is 4.51 (normal, 0.8–1.24), partial prothrombin time is 39.30S(normal range, 22–38S), thrombin time is 15.30S (normal range, 10–21S).

**Figure 1.**
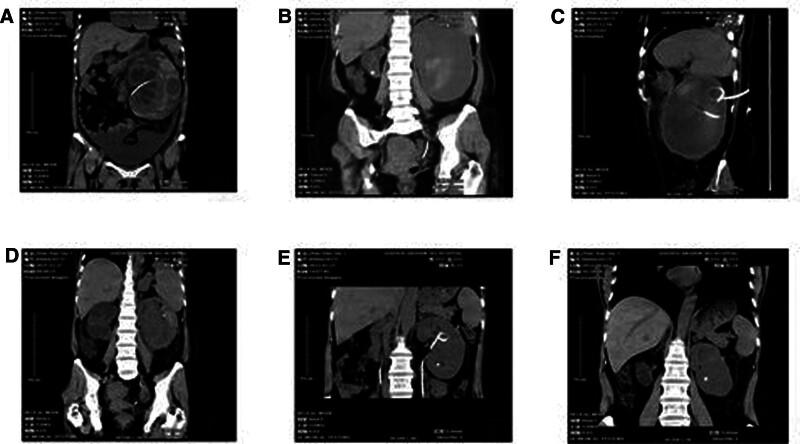
CT images of the patient during treatment. (A) The left ureteral stent was placed. (B) After the patient returned to our hospital, our reexamination of the CT scan showed that the perirenal hematoma had worsened compared to before and there was evidence of urinary extravasation. (C) The reexamination showed the situation of 2 drainage tubes. (D) Following placement of the nephrostomy tube, the patient had a fever and a reexamination CT showed hydrops in the right kidney. (E) The CT scan showed a significant reduction to hematoma and urine extravasation before removing Bilateral ureteral stents. CT = computed tomography.

To address the patient condition, we provided fresh frozen plasma and vitamin K to correct coagulation function, then inputted concentrated red blood cells to correct anemia, some drugs were given to stop bleeding and improve kidney function, we also given antibiotics to prevent infection. Then, the left ureteral stent was placed on November 5, 2021, a post-surgery CT scan was performed (Fig. 1A). Laboratory test results showed that renal function was normal, but coagulation function was still abnormal, we consider the possibility of hemophilia. By further laboratory examination, she was diagnosed with hemophilia (acquired hemophilia-factor VII deficiency). Our reexamination of the CT scan showed that the perirenal hematoma and urinary extravasation had worsened compared to before (Fig. 1B). We gave recombinant human coagulation factor VII to the patient by injection, but the patient experienced repeated fever with a body temperature of 39°C. We considered the possibility of infection for urine extravasation, and then performed left perirenal puncture stomy under local anesthesia on December 10, 2021 (Fig. 1C). The patient fever was under control 2 days after surgery. However, on December 26, the patient developed again with a body temperature of 39.1°C. The laboratory test showed a high white blood cell count, the reexamination of upper abdominal CT indicated that the scope left renal hematoma and urine extravasation were smaller than before, but there was the right renal hydronephrosis (Fig. 1D), the fever was considered to be caused by obstruction of the right kidney. The patient was placed on a right ureteral stent on December 27, 2021, and anti-infection treatment was continued provision after surgery. As a result, the patient condition has stabilized and was discharged from hospital.

The patient was regularly given coagulation factor in the outpatient department, and the perirenal tubes were pulled out during the outpatient follow-up in our department. The patient returned to our hospital on February 23, 2022, the CT scan showed a significant reduction to hematoma and urine extravasation (Fig. 1E), then Bilateral ureteral stents were removed. The patient was regularly followed up in the outpatient department of our hospital until complete absorption of the left renal hematoma on September 5, 2022 (Fig. 1F).

## 3. Discussion

Spontaneous renal rupture usually occurs after upper urinary calculi-related operation treatment or renal tumor,^[2,4]^ it has also been reported during pregnancy,^[5]^ transplant kidney and nephritis.^[6,7]^ According to the location of renal rupture, it can be divided into renal parenchyma rupture, collecting system rupture or mixed rupture,^[1]^ patient may experience corresponding symptoms, such as pain, hematuresis and Wunderlich syndrome.^[7]^ The patient did not have any obstruction and tumor, but this patient has a compensatorily enlarged left kidney due to the atrophy of the right kidney, this left kidney may be relatively fragile, more importantly, the patient has hemophilia, which is a bleeding-prone disease for the lack of clotting factor. She presented with gross hematuria and perirenal hematoma, these symptoms indicate that the left kidney is a mixed rupture.

The treatment method of spontaneous renal rupture is based on the underlying etiology and the hemodynamic state of the patient. When a patient hemodynamics is unstable, this may involve massive rehydration and blood transfusions for antishock therapy. In some cases, surgical exploration may be necessary to assess the extent of renal rupture and determine the need for repair or resection. For patients with stable hemodynamics, we recommend conservative treatment, which includes anti-infection therapy and symptomatic treatment. Conservative treatment is generally not effective in the case of a large amount of urinary extravasation, because prolonged extravasation can lead to liquefaction and formation of fibrous tissues which forms the perirenal pseudocyst.^[8]^ Bleeding may also lead to upper urinary tract obstruction, the combination of the 2 reasons will aggravate infection and impair renal function. In this situation, it is necessary to place a ureteral stent or a fistula to fully drain urine and keep urinary tract open, this can reduce the pressure on the renal system and relieve the pain symptoms of the patient. In addition, renal artery embolization should be considered for hemostasis.^[9]^

In this case report, there was also a limitation that the CT examination images of the patient prior to admission could not be presented, due to the short time interval and to reduce his financial burden, we did not reexamine CT scan as we only had film from the previous hospital. When spontaneous renal rupture is associated with abnormal coagulation function, and the coagulation function cannot be corrected by conventional treatment, the possibility of hemophilia needs to be considered, and the type of hemophilia needs to be further defined. Based on the diagnosis and treatment experience from this case. We hope this case can provide some reference value for clinical work.

## Author contributions

**Writing – original draft:** Chao Yang.

**Writing – review & editing:** Mingjun Luo, Langlang Li, Qizhi Yang.
